# Evaluation of in vitro anti-inflammatory and antibacterial potential of *Crescentia cujete* leaves and stem bark

**DOI:** 10.1186/s13104-015-1384-5

**Published:** 2015-09-04

**Authors:** Mst. Shahnaj Parvin, Nandita Das, Nusrat Jahan, Most. Afia Akhter, Laizuman Nahar, Md. Ekramul Islam

**Affiliations:** Department of Pharmacy, University of Rajshahi, Rajshahi, 6205 Bangladesh

**Keywords:** Anti-inflammatory, Antibacterial, Total phenol, Leaves, Stem bark

## Abstract

**Background:**

The various parts of *Cresecentia cujete* have some important biological activities. In folklore medicine leaves are used to treat hematomas, tumors and hypertension. Fruit decoction is used to treat diarrhea, stomachaches, cold, bronchitis, cough, asthma, and urethritis. The present study was designed to explore the anti-inflammatory and antibacterial potential of *C. cujete* leaves and stem bark. Anti-inflammatory activity was evaluated by in vitro human red blood cell (HRBC) membrane stabilization method and antibacterial activity by disc diffusion method.

**Methods:**

In vitro anti-inflammatory activity was evaluated by human red blood cell (HRBC) membrane stabilization method while in vitro antibacterial activity was evaluated using cultures of *Escherichia coli* and *Staphylococcus aureus* by disc diffusion method. Total phenolic (TPC) and total flavonoid contents (TFC) of the crude extract and fractions were also determined by Folin–Ciocalteu’s phenol reagent and by aluminium chloride method, respectively.

**Results:**

The crude ethanol extract (CEE) of leaves and bark (concentration of each 1.0 mg/ml) demonstrated strong membrane stabilizing activity (53.86 and 61.85 % protection, respectively), whereas their chloroform fractions (CHF) revealed moderate activity (48.74 ± 0.56 and 43.55 ± 6.20 %, respectively) compared with standard aspirin (concentration 0.10 mg/ml) which showed 75.81 % protection in this test. All the samples showed a dose dependent anti-inflammatory activity in HRBC membrane stabilization test. Total phenolic (TPC) and total flavonoid contents (TFC) of the crude extract and fractions were also determined. Again, in in vitro antibacterial study, the extractives exhibited potent antibacterial activity.

**Conclusion:**

Results from this study showed that the leaves and bark of *C. cujete* possessed anti-inflammatory as well as antibacterial activities indicating that the plant extract has therapeutic potential against the bacterial infection and also have effect on disease processes by causing destabilization of biological membranes.

## Background

Disease has been an integral part of man from the beginning of his existence. The subject of drugs is also as old as disease and the search for remedies to combat it is perhaps equally old and for more than a millennium, herbal medicine has been extensively used, apparently safely and effectively to alleviate various symptoms of disease [1]. The most important bioactive compounds of plants are alkaloids, flavonoids, tannins, glycosides and phenolic compounds [2, 3]. These compounds possess numerous health-related effects such as antibacterial, antimutagenic, anticarcinogenic, antithrombotic and vasodilatory activities [4].

Inflammatory diseases including different types of rheumatic diseases are very common throughout the World [5]. Although, the rheumatism is one of the oldest known diseases of mankind and affects a large population of the world and no substantial progress has been made in achieving a permanent cure. Non-steroidal anti-inflammatory drugs (NSAIDs) are used throughout the world for the treatment and management of inflammation, pain and fever. The use of NSAIDs, however, has not been therapeutically successful in all conditions of inflammation [6]. Moreover, adverse effects associated with NSAIDs can lead to ulcers and hemorrhage.

The expanding bacterial resistance to antibiotics has become a growing concern worldwide [7]. Intensive care physicians consider antibiotic-resistant bacteria a significant or major problem in the treatment of patients [8]. Increasing bacterial resistance is a prompting resurgence in research of the antimicrobial role of herbs against resistant strains [9]. A vast number of medicinal plants have been recognized as valuable resources of natural antimicrobial compounds [10].

As an alternative, plant based medicines are getting an increased therapeutics market share due to their mild action and fewer adverse effects. According to the World Health Organization nearly 80 % of the world population prefers plant based drugs [11]. The research on screening and development of drugs for their activity is therefore, an unending process and there is hope of finding out anti-inflammatory drugs from indigenous plants. Various plant extracts and their isolated compounds have been proved as good as synthetic anti-inflammatory agents [12]. Latest and previous studies have concluded the beneficial aspects of plant derived drugs as good source of antibiotics, antioxidants and anti-inflammatory agents [13, 14]. The present study was designed to investigate the TPC and TFC and to evaluate the anti-inflammatory and antibacterial activities of ethanol extract and fractions of leaves and bark of *C. cujete.*

*C. cujete* tree belongs to the family of Binoniacea. It is also known as the gourd tree or calabash tree. The tree is about 6–10 m tall with a wide crown and long branches covered with clusters of tripinnate leaves and gourd-like fruit. The branches have simple elliptical leaves clustered at the anode. According to folk medicine, the fruit pulp is used for respiratory problems such as asthma and also used as laxative. The bark is used for mucoid diarrhea. Bark decoction is used to clean wounds and pounded leaves are used as poultice for headaches. Internally, the leaves are used as diuretic and in the treatment of hematomas and tumors. Fruit decoction is used to treat diarrhea, stomachaches, cold, bronchitis, cough, asthma, and urethritis. The leaves on the other hand are used for the treatment of hypertension [15]. The juice from fruits mixed with sugar and/or bee’s honey are eaten for the purpose of solving the problems of the respiratory system (asthma, catarrh), the digestive system (stomach pains, intestinal parasites) and female reproductive apparatus (infertility) [16]. DPPH radical scavenging, antioxidant activity by β-carotene bleaching test and cytotoxic activity of the methanol extract of aerial parts of this plant were evaluated by Juceni et al. [17]. It has been reported that ethanol extract of *C. cujete* leaves showed significant antibacterial activity against *Shigella dysenteriae*, *Bacillus subtilis*, *Bacillus cereus*, *Bacillus megaterium* and *Staphylococcus aureus* [18]. However, to the best of our knowledge, no detailed anti-inflammatory and antibacterial activities on different fractions of leaves and bark extracts have been studied, yet. Hence, effort is being made here to investigate the crude ethanol extract and its chloroform fraction of *C. cujete* leaves and stem bark for in vitro anti-inflammatory and antibacterial activity.

## Methods

### Chemicals

Gallic acid and Folin-Ciocalteu were purchased from Sigma-Aldrich USA and Merck (Damstadt, Germany) respectively. Potassium ferricyanide, potassium acetate, phosphate buffer, catechin (CA), ferrous ammonium sulphate, ascorbic acid (AA), aluminium chloride (AlCl_3_), Trichloro acetic acid (TCA), sodium phosphate, ammonium molybdate, tannic acid, quercetin (QU), ethylenediamine tetra acetic acid (EDTA), acetyl acetone and ferric chloride (FeCl_3_) were purchased from Sigma Chemical Co. (St. Louis, MO, USA).

### Plant material

*C. cujete* leaves and bark samples were collected from University of Rajshahi, Rajshahi, Bangladesh. Identification of the plant was confirmed at the Department of Botany, University of Rajshahi and a voucher specimen (BG No. 534) has been deposited in the departmental herbarium. The collected plant parts were dried for 1 week and pulverized into a coarse powder using a suitable grinder. The powder was stored in an airtight container and kept in a cool, dark, and dry place.

### Extract preparation

Approximately 450 g of powdered leaves and 380 g of powdered bark was placed separately in a clean, flat-bottomed glass container and soaked in ethanol. The container with its contents was sealed and kept for 7 days accompanied by occasional shaking and stirring. The entire mixture then underwent a coarse filtration by a piece of clean, white cotton material. The extract then was filtered through Whatman filter paper (Bibby RE200, Sterilin Ltd., UK). After filtration, the filtrate was evaporated to dryness at 50 °C under reduced pressure using a rotary evaporator to obtain the ethanol crude extract (9.5 g for leaves and 8.5 g for bark). The crude ethanol extract (CEE) was suspended with distilled water (150 ml) and partitioned with petroleum ether and chloroform. Here, crude ethanol extract (CEE) and chloroform fraction (CHF) were used for the biological screenings.

### Determination of total phenolic content

The concentrations of phenolic compounds in the samples of *C. cujete* leaves and bark were measured according to the Folin-Ciocalteu method [19]. Briefly, the samples solution (0.5 ml) at different concentrations (ranging from 100 to 1100 μg/ml) was mixed with 2.58 ml of Folin–Ciocalteu’s phenol reagent. After 3 min, 0.3 ml of saturated sodium carbonate solution was added to the mixture. The reaction mixtures were incubated at room temperature (25 °C) for 20 min. The absorbance was measured at 760 nm with a spectrophotometer. Gallic acid solutions with concentrations ranging from 25 to 400 μg/ml were used for calibration. A dose response linear regression was generated by using the gallic acid standard absorbance and the levels in the samples were expressed as gallic acid equivalent (mg of GAE/gm of extract). The estimation was performed in triplicate, and the results were expressed as mean ± SD.

### Determination of total flavonoid content

The total flavonoid content was estimated by aluminium chloride method [20]. Plant samples (0.5 ml) were mixed with 2.5 ml of distilled water and 150 μl NaNO2 solution (5 %). The contents were vortexed for 10 s and left at room temperature for 5 min. Then, 300 μl AlCl3 (10 %), 1 ml NaOH (1 mM) and 550 μl of distilled water were added. The solution was mixed well and kept for 15 min. The absorbance for each sample was measured at 510 nm. Quercetin concentrations ranging from 25 to 400 μg/ml were prepared and the standard calibration curve was obtained. The total flavonoid content was calculated using standard quercetin calibration curve. The results were expressed as mg of quercetin equivalent (QE) per gram of extract.

### Anti-inflammatory activity

#### Preparation of blood samples for membrane stabilization assays

The human red blood cell (HRBC) membrane stabilization method has been used as a method to study the in vitro anti-inflammatory activity [21]. The blood was collected from healthy human volunteer who had not taken any NSAIDS for 2 weeks prior to the experiment and mixed with equal volume of Alsever solution(2 % dextrose, 0.8 % sodium citrate, 0.5 % citric acid and 0.42 % NaCl). All the blood samples were stored at 4 °C for 24 h before use. It was centrifuged at 2500 rpm for 5 min and the supernatant was removed. The cell suspension was washed with sterile saline solution (0.9 % w/v NaCl) and centrifuged at 2500 rpm for 5 min. This was repeated three times till the supernatant was clear and colorless and the packed cell volume was measured. The cellular component was reconstituted to a 40 % suspension (v/v) with phosphate buffered saline (10 mM, pH 7.4) and was used in the assays.

#### Hypotonicity-induced haemolysis

CCE and CHF of *C. cujete* leavesand bark were prepared (100 and 1000 µg/ml), respectively using distilled water and to each concentration 1 ml of phosphate buffer, 2 ml hyposaline and 0.5 ml of HRBC suspension were added. It was incubated at 37 °C for 30 min and centrifuged at 3000 rpm for 20 min. The haemoglobin content of the supernatant solution was estimated spectrophotometrically at 560 nm. Aspirin (100 µg/ml) was used as reference standard and a control was prepared by omitting the extracts. The percentage inhibition of haemolysis or membrane stabilization was calculated according to modified method described by Shinde et al. [22].

$$\% {\text{ Inhibition of haemolysis }} = { 1}00 \, \times \, \left\{ {{\text{OD1}} - {\text{OD2}}/{\text{OD1}}} \right\}$$

where: OD1 = Optical density of hypotonic-buffered saline solution alone.OD2 **=** Optical density of test sample in hypotonic solution.

#### Antibacterial assay

The antimicrobial activity of ethanol crude extract and chloroform fractions of leaves and bark were screened at two concentrations (100 and 200 μg/disc) against *Staphylococcus aureus* (Gram-positive) and *Escherichia coli* (Gram-negative) using the disc diffusion method [23]. The organisms were collected from the Microbiology Research Laboratory, Department of Pharmacy, Rajshahi University, Bangladesh. Solutions of known concentration (10 mg/ml) of the test samples were prepared by dissolving measured amounts of samples in calculated solvent volumes. Dried and sterilized filter paper discs (6-mm diameter) were then impregnated with known amounts of the test substances using a micropipette. Discs containing the test material were placed on nutrient agar medium (Merck) uniformly seeded with the pathogenic test microorganisms. Antibiotic Kanamycin-K (25 μg/disc) and blank discs (impregnated with solvents) were used as positive and negative controls, respectively. These plates were then, kept at 4 °C for a 1-h diffusion of the test material. There was a gradual change in concentration surrounding the discs. The culture plates were then, incubated at 37 °C for 24 h to allow organism growth. The test materials having antibacterial activity inhibited microorganism growth, and a clear, distinct zone of inhibition surrounding the discs was visualized. The antibacterial activity of the test agents was determined by measuring the diameter of the zone of inhibition expressed in millimeters.

### Ethic’s statement

The experimental procedure involving human RBC was approved by the Institutional Animal, Medical Ethics, Biosafety and Biosecurity Committee (IAMEBBC) for Experimentations on Animal, Human, Microbes and Living Natural Sources (225/337-IAMEBBC IBSc), Institute of Biological Sciences, University of Rajshahi, Bangladesh.

### Consent statement

Participation was voluntary and written informed consent was taken from all the respondents after fully explaining the nature and purpose of, and all procedures used for the study.

### Statistical analysis

The statistical analyses were performed by a one-way ANOVA and the Student’s *t* test. Free Rsoftware version 2.15.1 (http://www.r-project.org/) and Microsoft Excel 2007 (Roselle, IL, USA) were used for the statistical and graphical evaluations. The results were expressed as mean ± SD from three separate observations.

## Results

### Total phenolic content

**Fig. 1:**
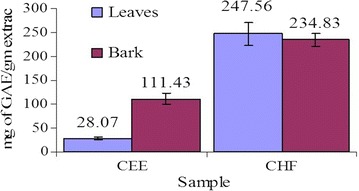
Total phenolic content of leaves and bark. Values are 388 represented as mg of GAE/g of extract. *Each value* in the graph is represented as mean ± SD (n = 3)

The TPC of the tested samples were shown in Fig. 1. The samples contained a considerable amount of phenolic compounds. TPC expressed in terms of GAE of CEE and CHF of *C. cujete* leaves were 28.07 ± 9.47 and 247.56 ± 24.06 mg of GAE/g of extract, respectively; whereas that of bark were 111.43 ± 10.65 and 234.83 ± 13.15 mg of GAE/g of extract, respectively. TPC were calculated using the following linear equation based on the calibration curve of gallic acid; y = 0.0068x + 0.2719, R^2^ = 0.9715.

### Total flavonoid content

**Fig. 2:**
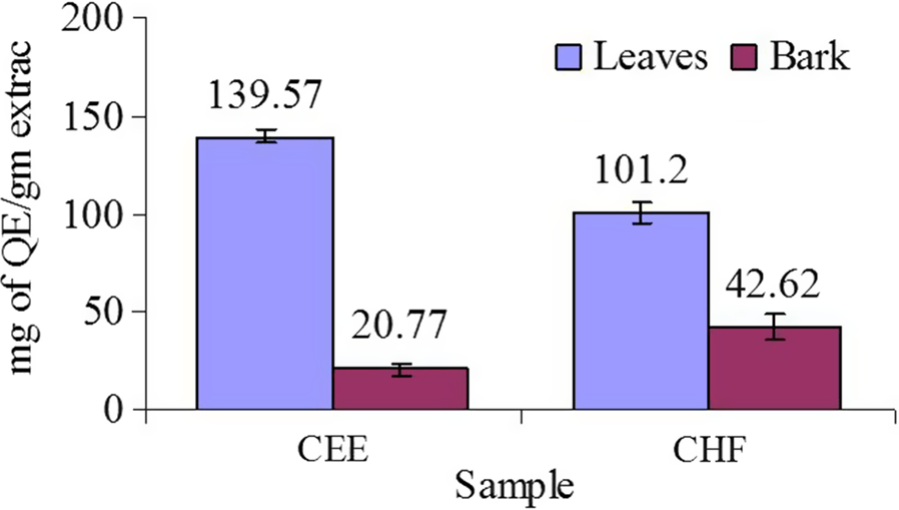
Total flavonoid content of leaves and bark. Values are represented as mg of QE/g of extract. *Each value* in the graph is represented as mean ± SD (n = 3)

TFC was expressed as mg of quercetin equivalents per gm of dry extract. Flavonoid contents varied widely among CEE and CHF of leaves and bark. CEE of leaves exhibited highest flavonoid contents (139.57 ± 3.75 mg of QE/gm of extract) followed by CHF of leaves, CHF and CEE of bark in the decreasing order (Fig. 2). TFC in the samples were determined with reference standard curve of quercetin (y = 0.0062x + 0.0039, R^2^ = 0.932).

### Anti-inflammatory activity

**Fig. 3:**
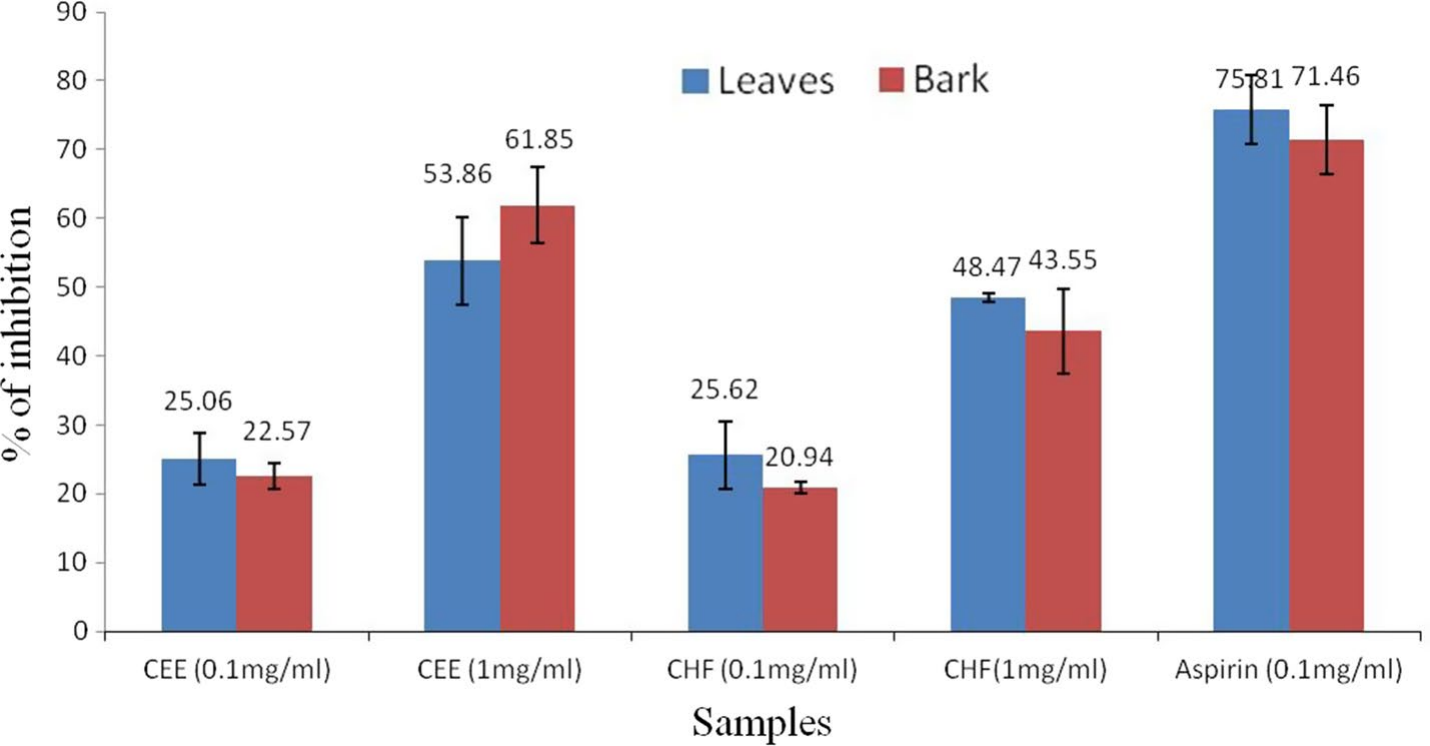
Effect of CEE and CHF of *C. cujete* leaves and stem bark on hypotonic solution-induced hemolysis of erythrocyte membrane. *Each value* is expressed as mean ± SD (n = 3). *CEE* crude ethanol extract, *CHF* chloroform fraction

The results of anti-inflammatory activity determined by the human red blood cell membrane stabilization test were shown in Fig. 3. The CEE and CHF of leaves and bark showed a concentration dependent anti-inflammatory activity, and the protection percent increased with increase in the concentration of the samples. At concentration of 1.0 mg/ml, the CEE of leaves and bark produced 53.86 ± 6.37 and 61.85 ± 5.56 % inhibition of RBC hemolysis, respectively as compared with 75.80 ± 5.04 % produced by standard drug aspirin. Likewise, CHF of leaves and bark produced 48.74 ± 0.56 and 43.55 ± 6.20 % inhibition of RBC hemolysis, respectively. It is clear from the data that CEE of leaves and bark showed grater response than the CHF.

### Antimicrobial properties

Table 1 showed the antimicrobial activities of extract/fraction of *C. cujete* leaves and bark against tested bacterial strains. Their antibacterial potency was assessed by the presence or absence of a zone of inhibition and zone diameters (mm). The CHF of both leaves and stem bark exhibited better antimicrobial activity against the tested organisms. The CEE of stem bark exerted the lowest activity against *E. coli*. The maximum zone of inhibition was obtained for CHF of leaves at 200 μg/disc against *E. coli*.

**Table 1:** In vitro antibacterial activity of CEE and CHF of *Crescentia cujete* leaves and stem bark

Test sample	Concentrations (µg/disc)	Test organisms and their of zone of inhibition in diameter (mm)	
		*Staphylococcus aureus*	*Esherichia coli*
Leaves			
CEE	100	8	11
	200	10	18
CHF	100	14	10
	200	15	29
Stem bark			
CEE	100	NS	5
	200	NS	8
CHF	100	11	9
	200	11	10
Kanamycin-K	30	21	27

*NS* no sensitivity

## Discussion

The successive leaves and stem brak extract of *C. cujete* exhibited membrane stabilization effect by inhibiting hypotonicity induced lysis of erythrocyte membrane. The erythrocyte membrane is analogous to the lysosomal membrane and its stabilization implies that the extract may as well stabilize lysosomal membranes [24]. Stabilization of lysosomal membrane is important in limiting the inflammatory response by preventing the release of lysomal constituents of activated neutrophil such as bactericidal enzymes and proteases, which cause further tissue inflammation and damage upon extra cellular release [25]. Though the exact mechanism of the membrane stabilization by the extract is not known yet, hypotonicity induced hemolysis may arise from shrinkage of the cells due to osmotic loss of intracellular electrolyte and fluid components. The extract may inhibit the processes, which may stimulate or enhance the efflux of these intracellular components [26, 27]. On the basis of in vitro evaluated results CHF showed significant anti-inflammatory activity as compared to control. Anti-inflammatory effects have been observed in flavanoids as well as tannins. Flavonoids such as quercetin are known to be effective in reducing acute inflammation. Certain flavonoids possess potent inhibitory activity against a variety of enzymes such as proteinkinase C, protein tyrosine kinases, phospholipase A_2_, phosphodiesterases. The anti-inflammatory activity of the extract/fraction may be due to the presencre of flavanoids, tannins etc. either singly or in combination [28]. In vitro result suggests that the leaf extract of *C. cujete* possess potential anti-inflammatory activity.

For the antibacterial activity study, the extracts/fractions of leaves and stem bark of *C. cujete* displayed activity against two pathogenic bacteria to different magnitudes. CHF of leaves possessed the greatest antimicrobial effects. The phenolics and polyphenols are one of the largest groups of secondary metabolites that have exhibited antimicrobial activity. Phenols are a class of chemical compounds consisting of a hydroxyl functional group (-OH) attached to an aromatic phenolic group. The site(s) and number of hydroxyl groups on the phenol group are thought to be related to their relative toxicity to microorganisms. They cause hyper acidification at the plasma membrane interface of the pathogen, which potentially results in the disruption of the H^+^-ATPase required for ATP synthesis [18] reported on the antibacterial activity of ethanolic extract of leaves against *Shigella dysenteriae*, *Bacillus cereus*, *Bacillus subtilis*, *Bacillus megaterium* and *Staphylococcus aureus.* On the other hand, flavonoids are also hydroxylated phenolic substances but occur as a C6-C3 unit linked to an aromatic ring. Flavones, flavonoids and flavonols have been known to be synthesized by plants in response to microbial infection so it is not surprising that they have been found, in vitro, to be effective antimicrobial substances against a wide array of microorganisms [29]. Their activity is probably due to their ability to complex with extracellular and soluble proteins and to complex with bacterial cell walls. In our study CHF of leaves showed the highest activity against *E. coli* with a zone of inhibition 29 mm which was found equivalent to standard disc of Kanamycin K 30 µg/disc. The organisms that were susceptible to the *C. cujete* leaves and stem bark extracts may contain the active compounds of the above classes that can inhibit the proliferation and growth of these organisms. Since, the compounds and mechanisms of action responsible for the antibacterial activities of these extracts are currently unclear; further studies are necessary in order to isolate and identify some active compounds which may be responsible for the activity and to explore the mechanism of action of *C. cujete* leaves and bark extracts.

## Abbreviations

*C. cujete*: *Cresecentia cujete*
HRBC: human red blood cell
TPC: total phenolic and
TFC: total flavonoid contents
CEE: crude ethanol extract
CHF: chloroform fractions
NSAIDs: non-steroidal anti-inflammatory drugs
DPPH: 2,2-diphenyl-1-picrylhydrazyl
CA: catechin
AA: ascorbic acid
TCA: trichloro acetic acid
EDTA: ethylenediamine tetra acetic acid
GAE: gallic acid equivalent
QE: Quercetin equivalent
NS: no sensitivity
